# Early neurological deterioration in acute lacunar ischemic stroke: Systematic review of incidence, mechanisms, and prospects for treatment

**DOI:** 10.1177/17474930241273685

**Published:** 2024-09-05

**Authors:** David J Werring, Hatice Ozkan, Fergus Doubal, Jesse Dawson, Nick Freemantle, Ahamad Hassan, Suong Thi Ngoc Le, Dermot Mallon, Rom Mendel, Hugh S Markus, Jatinder S Minhas, Alastair J S Webb

**Affiliations:** 1 Stroke Research Centre, UCL Queen Square Institute of Neurology, London, UK; 2 Centre for Clinical Brain Sciences, Row Fogo Centre for Research into Ageing and the Brain, University of Edinburgh, Edinburgh, UK; 3 School of Cardiovascular and Metabolic Health, University of Glasgow, Glasgow, UK; 4 Comprehensive Clinical Trials Unit, University College London, London, UK; 5 Department of Neurology, Leeds General Infirmary, Leeds, UK; 6 Lysholm Department of Neuroradiology, National Hospital for Neurology and Neurosurgery, London, UK; 7 Department of Clinical Neurosciences, Stroke Research Group, University of Cambridge, Cambridge, UK; 8 NIHR Leicester Biomedical Research Centre, Department of Cardiovascular Sciences, University of Leicester, Robert Kilpatrick Clinical Sciences Building, Leicester, UK; 9 Imperial College London, London, UK

**Keywords:** Acute stroke therapy, stroke, lacunar stroke, small vessel disease, cerebral perfusion, antithrombotic

## Abstract

**Background:**

Cerebral small vessel disease (CSVD) causes between 25% and 30% of all ischemic strokes. In acute lacunar ischemic stroke, despite often mild initial symptoms, early neurological deterioration (END) occurs in approximately 15–20% of patients and is associated with poor functional outcome, yet its mechanisms are not well understood.

**Aims:**

In this review, we systematically evaluated data on: (1) definitions and incidence of END, (2) mechanisms of small vessel occlusion, (3) predictors and mechanisms of END, and (4) prospects for the prevention or treatment of patients with END.

**Summary of review:**

We identified 67 reports (including 13,407 participants) describing the incidence of END in acute lacunar ischemic stroke. The specified timescale for END varied from <24 h to 3 weeks. The rate of END ranged between 2.3% and 47.5% with a pooled incidence of 23.54% (95% confidence interval (CI) = 21.02–26.05) but heterogeneity was high (*I*^2^ = 90.29%). The rates of END defined by National Institutes of Health Stroke Scale (NIHSS) decreases of ⩾1, ⩾2, ⩾3, and 4 points were as follows: 24.17 (21.19–27.16)%, 22.98 (20.48–25.30)%, 23.33 (16.23–30.42)%, and 10.79 (2.09–23.13)%, respectively, with lowest heterogeneity and greatest precision for a cutoff of ⩾2 points. Of the 20/67 studies (30%) reporting associations of END with clinical outcome, 19/20 (95%) reported worse outcomes (usually measured using the modified Rankin score at 90 days or at hospital discharge) in patients with END. In a meta-regression analysis, female sex, hypertension, diabetes, and smoking were associated with END.

**Conclusions:**

END occurs in more than 20% of patients with acute lacunar ischemic stroke and might provide a novel target for clinical trials. A definition of an NIHSS ⩾2 decrease is most used and provides the best between-study homogeneity. END is consistently associated with poor functional outcome. Further research is needed to better identify patients at risk of END, to understand the underlying mechanisms, and to carry out new trials to test potential interventions.

## Background

Cerebral small vessel disease (CSVD) causes between 25% and 30% of all ischemic strokes;^1^ these strokes are due to a small, subcortical infarct resulting from occlusion of a small perforating artery, leading to characteristic clinical syndromes including pure motor hemiparesis, pure sensory stroke, ataxic hemiparesis, dysarthria-clumsy hand syndrome, and sensorimotor stroke.^2^ These clinical patterns are often termed “lacunar syndromes” due to their associations with lacunes, the chronic cavitated lesions that frequently develop from small subcortical infarcts over time. Here, for convenience and brevity, we will use the term “acute lacunar ischemic stroke” to refer to a clinical syndrome suggesting stroke associated with a recent small subcortical infarction consistent with occlusion of a small perforating artery.^3^

By contrast with ischemic stroke due to large vessel occlusion, which can now be effectively treated with mechanical thrombectomy, there are no specific treatments for acute lacunar ischemic stroke. A *post hoc* subgroup analysis of the WAKE-UP trial showed functional benefit from alteplase in patients who had stroke symptoms either on awakening or of uncertain onset, who also had small subcortical infarction on diffusion-weighted magnetic resonance imaging (MRI),^4^ but systematic reviews have not definitively shown benefit for thrombolysis in acute lacunar ischemic stroke.^5^ A recent guideline concluded that data on alteplase in acute lacunar ischemic stroke showed confidence intervals (CIs) for benefit overlapping the line of no effect (albeit with a beneficial trend consistent with the overall acute stroke trial results).^6^ A lack of benefit from thrombolysis may be because small vessel occlusion is not necessarily due to thrombosis, but, rather, a “functional” occlusion of the vessel due to largely unknown mechanisms.

A characteristic feature of acute stroke due to small vessel occlusion is that, despite often mild initial symptoms, “early neurological deterioration” (END) can occur. This term describes a worsening of neurological function (typically motor deficits), usually in the hours or first day or two after symptom onset.^7^ END can occur in all stroke types,^8^ but has been reported to be more common in acute lacunar ischemic stroke than other stroke aetiologies,^9^ affecting approximately 15–20% of patients.^10^ Importantly, END is associated with increased disability after stroke and poorer functional outcomes at hospital discharge and longer-term follow-up,^7,11^ although the proportion of variation in functional outcome attributable to END—and that could potentially be treated—is not known. The mechanisms of END are not well understood, but may include altered perfusion, excitotoxity, inflammation, edema causing conduction block, or thrombus propagation.^12^ Understanding and preventing END in acute lacunar ischemic stroke is, therefore, an unmet important clinical need.

## Definitions of END in acute lacunar ischemic stroke

END is defined in a variety of ways, with different cutoffs for the degree of National Institutes of Health Stroke Scale (NIHSS) worsening (sometimes specifying a specific NIHSS change threshold for motor deterioration, but sometimes not) and time course (from 24 h to 3 weeks or during hospital admission). This heterogeneity in definitions makes comparisons between studies potentially challenging.

## Capsular warning syndrome

This syndrome consists of recurrent (“crescendo”) transient lacunar sensorimotor syndromes (i.e., episodes consistent with subcortical ischemia in the territory of a perforating artery supplying the internal capsule).^13^ The attacks (which are usually stereotyped and occur in a cluster of several (⩾3) to many in a short period of less than 24–72 h)^14^ cause weakness (with or without sensory disturbance) affecting the face, arm, and leg (usually all three regions, but sometimes fewer), without cortical features. The mechanism of fluctuation has been postulated to be hemodynamic, partly because there is no clear evidence of benefit from antiplatelet, anticoagulant, or thrombolytic treatments. The risk of infarction (and persistent neurological deficit) is high (42% in the original series^13^ and 71.2% in a more recent multicentre report).^14^ Structures other than the internal capsule which are supplied by perforating small arteries can also be affected, including the pons.^15^ Capsular warning syndrome is rare (1.5% of all transient ischemic attack (TIA) syndromes in one population-based study)^16^ but of mechanistic interest as it may shed light on the mechanisms underlying END in acute lacunar ischemic stroke.

### Mechanisms of acute small vessel occlusion

There are three main potential mechanisms postulated for acute lacunar ischemic stroke: first, small vessel occlusion due to *in situ* disease (arteriolosclerosis) of the small perforating vessel itself (causing occlusion through thrombosis or some other mechanism, discussed below); second, occlusion due to atheromatous disease affecting the parent artery where the small vessel originates (often termed “branch atheromatous disease” (BAD), Figure 3); and third, occlusion by embolism from another, more proximal, source, such as atherosclerotic stenosis of the extracranial vessels or a cardioembolic source (commonly atrial fibrillation). The relative frequency of these different mechanisms remains uncertain, but pathological studies and more recent imaging studies can shed light on this question.

#### In situ small vessel disease

C.M. Fisher, by undertaking careful dissection in four patients with a history of hypertension and stroke 2 months or more prior to death, described a process of “segmental arterial disorganization,” likely to be within the spectrum of small vessel pathological processes now termed arteriolosclerosis, lipohyalinosis, or fibrinoid necrosis.^2,17^ However, it remains uncertain how often occlusion of abnormal small perforating arteries is due to “true” thrombosis. In one MRI and computed tomography (CT) study, 9 of 80 patients (11.2%) had evidence of a linear structure with density or signal features consistent with an occluded (possibly thrombosed, but certainly abnormal) perforating artery associated with a lacunar infarct.^18^ The authors hypothesized that in some cases the appearance might have been caused by a leak of blood and fluid into the perivascular space around the artery, as in several patients the diameter of the observed tubular vessel-like structure (>1 mm) was greater than the expected width of a perforating artery (<0.8 mm); further support came from the location of infarction around, rather than at the end of, the abnormal vessel.^18^ These findings support Miller Fisher’s findings of local enlargement of the vessel due to focal hemorrhagic extravasation through the wall into the perivascular region;^18^ however, a major limitation of this study was the long delay to imaging after stroke onset (7–58 days), meaning that it is difficult to know whether the findings are relevant to initial (acute) vessel dysfunction or are secondary appearances. Surprisingly, few subsequent studies have confirmed or extended these interesting early findings, making this a topic for further research using modern MRI techniques. High-field (7T) MRI has potential to visualize individual perforating arteries to see whether there is thrombus or occlusion in flow in them, although undertaking such studies in the hyperacute phase of stroke is challenging.^19^

Underlying mechanisms postulated for abnormal leakage from small vessels include endothelial or blood–brain barrier (BBB) dysfunction. Endothelial dysfunction may also cause vasoconstriction with impaired autoregulation, leading to an inability to maintain perfusion distally,^20^ while loss of BBB integrity may cause local edema with plasma protein deposition in the vessel wall which could lead to stenosis or occlusion. Although widespread BBB leakage is established as a core feature of CSVD,^19^ the mechanisms underlying a sudden dysfunction in a single perforator remain uncertain; possible triggers could include antecedent infection, inflammation, acute systemic hypertension or hypotension, or spontaneous local vessel dysfunction, and require further study.

#### Branch Atheromatous Disease (BAD)

Intracranial BAD is characterized by the occlusion of a perforating branch (typically around 700–800 µm) near the orifice of a parent large artery due to atherosclerotic plaque (microatheroma)-associated thrombosis or thromboembolism^21^ (Figure 3(a)–(c)). BAD may be particularly relevant in Asian populations due to the high prevalence of intracranial atherosclerosis. The optimal definition of BAD remains uncertain, but the current consensus is that BAD can be inferred from brain and vessel imaging findings, for example the presence of a single subcortical infarction that is larger than typical lacunar infarction (the latter typically defined using an upper cutoff of 20 mm diameter) in the absence of visible parent stenosis of the major artery supplying the relevant deep perforating arteries.^22,23^ Other definitions include infarction on three or more horizontal scan slices in the lenticulostriate artery territory or infarction extending to the basilar pons in the paramedian pontine artery territory on diffusion-weighted imaging (DWI).^22^ The prevalence of BAD as a cause of acute lacunar ischemic stroke remains uncertain because it is not considered in most widely used stroke classification systems (e.g., TOAST). Nevertheless, in the large NAVIGATE-ESUS study, BAD was diagnosed in 502 (12.6%) out of 3972 patients (with a higher prevalence of 14.6% in East Asian countries compared to 9.3% in all other non-East Asian countries).^24^ Asian hospital-based studies (in Japan and Hong Kong) reported a comparable prevalence of 152 of 1665 (9.1%)^25^ and 132/720 (18.3%)^26^ patients with ischemic stroke. BAD may be relevant to interventions aimed at preventing recurrent events or clinical decline, with a potentially greater benefit of dual antiplatelet treatment in acute lacunar infarcts with BAD (due to reduced embolism from the parent vessel into the perforator) than without, although a subgroup analysis from the CHANCE trial did not support this hypothesis.^27^ In addition to the size of the acute infarct, the presence of BAD can also be inferred by the neuroimaging finding of “isolated” small subcortical infarction, without additional findings of CSVD (i.e., white matter hyperintensities and lacunes). Such isolated small subcortical infarcts have a different risk factor profile (being associated with hypercholesterolemia, diabetes, and myocardial infarction, consistent with an atherosclerotic cause)^28^ as well as a different genetic architecture.^29^

In this systematic review and meta-analysis, we evaluated data on (1) definitions and incidence of END in acute lacunar ischemic stroke; (2) mechanisms of small vessel occlusion; (3) predictors and mechanisms of END; and (4) prospects for the prevention or treatment of END. We discuss associations and mechanisms of END, and prospects for its prevention or treatment.

## Systematic review: methods

**Figure 1.**
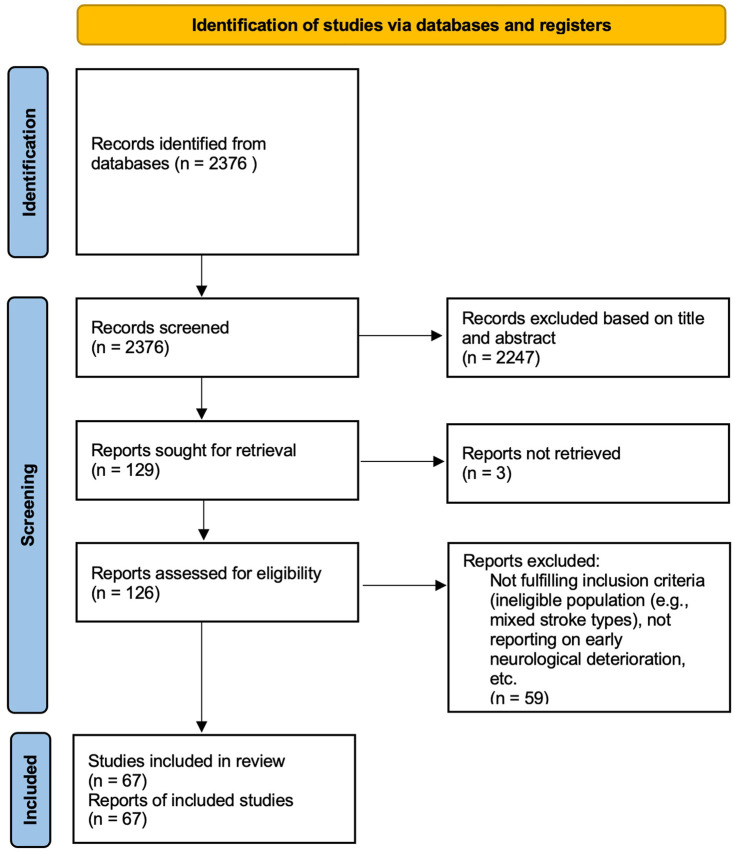
PRISMA flow diagram of included studies.

We followed Preferred Reporting Items for Systematic Reviews and Meta-Analyses (PRISMA) guidelines to search PubMed on 28 July 2023 (from inception) for articles in English describing END in patients with acute subcortical (lacunar) ischemic stroke. We did not specify a duration in which END should occur after stroke onset. The search structure was as follows: “(lacun* OR subcort* or “small vessel”) AND (deteriorat* OR worsen* OR progress* OR fluctuat*) AND (stroke OR cerebrovasc* OR infarct* OR occlu*). This identified 2376 references. After screening (by D.J.W.), we sought and retrieved 129 reports. After assessment for eligibility (by D.J.W.), we included 67 studies (including 13,407 participants) in our systematic review describing the incidence of END in acute subcortical infarction (Table 1). Data were extracted by D.J.W. and checked by H.O. The PRISMA flow chart is shown in Figure 1. We, in addition, found 10 references for the term “capsular warning syndrome.” We extracted population characteristics (including demographics and vascular risk factors) and the rate of END for each included study. We pooled data on the incidence of END and stratified according to each definition according to NIHSS score change criteria. We did a meta-regression analysis to investigate potential associations of study population factors with the incidence of END. All statistical analyses were performed by H.O. using STATA Version 18. We evaluated publication bias by creating a funnel plot.

**Table 1.** Baseline characteristics of patients in the included studies.

Characteristics	N = 67 studies (13,407 participants)
Infarct location	
Lenticulostriate or mixed lenticulostriate and pons	64 (95.5%)
Pons only	3 (4.48%)
Age	66 (5.3)
Female sex	27 (41%)
Systolic blood pressure median (IQR)^N^ ^=^ ^59^	155 (107–202)
Diabetes^N^ ^=^ ^*59*^	30 (51%)
Smoking^N^ ^=^ ^*55*^	37 (68%)
Total time to last reported clinical follow-up^N^ ^=^ ^*60*^	
48 h	10 (16.7%)
>7 days	16 (26.7%)
>10 days	2 (3.3%)
>20 days	32 (53.3%)

N *=* *number of studies for which data are reported.*

## Systematic review: results

**Figure 2.**
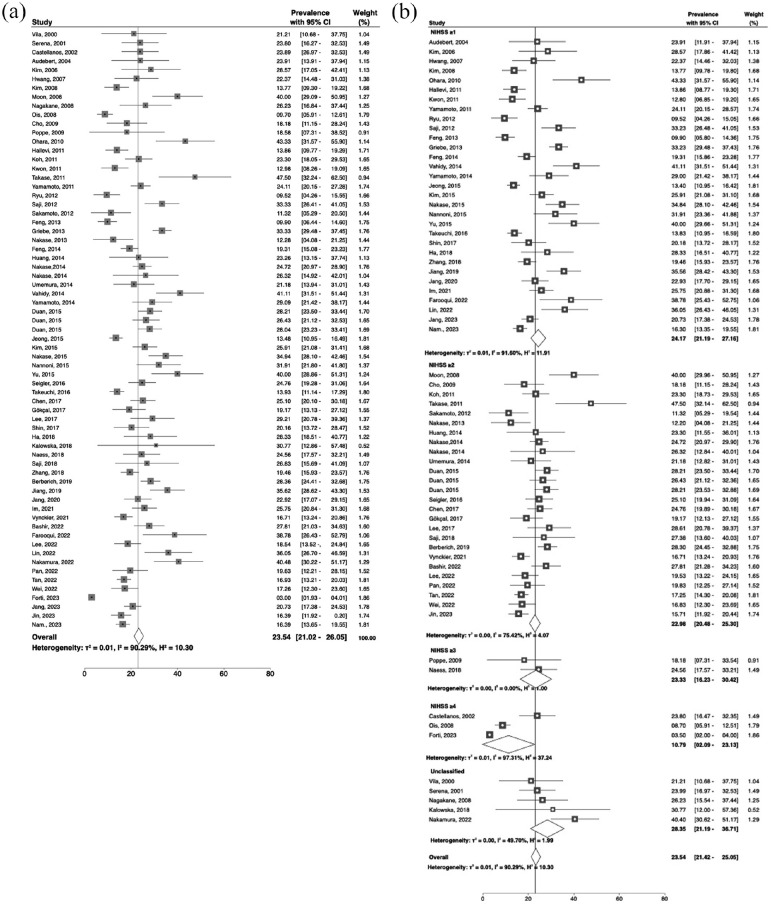
Forest plots showing the incidence of early neurological deterioration in acute lacunar ischemic stroke. (a) All studies, and (b) studies grouped by the NIHSS threshold for deterioration.

Information on the most consistently reported population characteristics in the included studies is provided in Table 1. The forest plot of the incidence of END found in all included studies is shown in Figure 2. The rate of END varied between 2.3% and 47.5% with a pooled incidence of 23.54% (95% CI = 21.02–26.05); heterogeneity was high (*I*^2^ = 90.29%) (Figure 2(a)). Reasons for the wide range in incidence estimates could include the varied definitions used for END, typically based on neurological function, most often measured with the NIHSS. Common definitions include an increase of ⩾1 or ⩾2 points on the NIHSS (Figure 2(b)). Some studies specify not only an overall NIHSS deterioration but also an additional minimum decline in the motor domain (e.g., a drop in ⩾2 NIHSS points but at least a ⩾1 point increase in the motor impairment score). Some studies used other instruments (e.g., Canadian Stroke Scale), precluding comparisons with NIHSS-based reports challenging. Another varying factor is the time window specified for deterioration, which varied from 24 h to 3 weeks or at any time during hospital admission. These factors increase heterogeneity, making harmonization across studies to investigate the incidence and predictive factors more challenging.

We identified 31 studies defining END after acute subcortical infarction with an NIHSS deterioration threshold of ⩾1, 26 with ⩾2, 2 with ⩾3, 3 with ⩾4, and 5 unclassified (Figure 2(b)). The incidence rates of END for NIHSS declines of ⩾1, ⩾2, ⩾3, and 4 points were 24.17 (21.19–27.16)%, 22.98 (20.48–25.30)%, 23.33 (16.23–30.42)%, and 10.79 (2.09–23.13)%, respectively (Figure 2(b)); heterogeneity was lower, and precision greater (narrower 95% CI) for a cutoff of NIHSS ⩾2 points than for the others.

The funnel plot to assess publication bias is shown in Supplemental Figure 1. A detailed description of all studies identified is shown in Supplemental Table 1. Only 17/67(25%) described the time course of END; moreover, the specified timescale within which to measure END varied widely from <24 h to 3 weeks (or, in some cases, reported at any time during hospital admission). Nevertheless, where reported, most deterioration occurred early, in the first 24 h: two studies described deterioration in the first 24 h in 9/11 (82%)^30^ and 15/17 (88%) of patients,^31^ respectively, while another study reported median and average times to END of 21.5^32^ and 24 h,^33^ respectively. Even in studies where a later time for END is described the vast majority appears to occur in the first 48–72 h after symptom onset. Of the 20/67 studies (30%) reporting associations of END with clinical outcome, 19/20 (95%) reported worse outcomes (usually measured using the modified Rankin score at 90 days or at hospital discharge) in patients with END (Supplemental Table 1).

In a meta-regression analysis to investigate potential associations of study population factors (Table 2), we found that female sex, hypertension, diabetes, and smoking were associated with END.

**Table 2.** Meta-regression analysis of reported study population characteristics associated with early neurological deterioration in acute lacunar ischemic stroke.

Study level characteristic	Adjusted odds ratio (aOR) (95% CI)
Infarct location	0.98 (0.56–1.54), p = 0.369
Age	0.87 (0.62–1.07), p = 0.121
Female sex	4.27 (2.17–6.63), p < 0.001
Hypertension	2.05 (1.34–3.48), p = 0.005
Diabetes	3.23 (1.21–4.09), p < 0.001
Smoking	4.06 (2.72–6.42), p = 0.013

A description of associations with END from the studies identified in our systematic review is shown in Table 3. To simplify these data, we have grouped the reported associations with END into the following categories: features of the acute infarct (morphology, location, size, growth, clinical severity); branch or parent atheromatous disease; hemodynamic or autonomic abnormalities; CSVD burden; markers of systemic inflammation; and glucose or lipid pathway abnormalities.

**Table 3.** Summary of the most frequently reported associations with early neurological deterioration in acute lacunar ischemic stroke.

**Features of the infarct (morphology, location, size, growth, clinical severity)** ** • Proximal location** ** • Increase in lesion volume or large DWI lesion** ** • NIHSS score on admission** (*likely correlated with lesion size, location, or both)* • Speckled DWI lesion • Irregular infarct shape • Conglomerated beads shape • Satellite lesions • Proximal pattern • Involvement of the corticospinal tract, or corona radiata adjacent to lateral ventricle • Length of infarcted tissue **Branch or parent atheromatous disease** **Hemodynamic or autonomic abnormalities** ** • Reduced perfusion** ** • Hypertension on admission** ** • Pulsatility index, arterial stiffness** • Impaired sympathetic function • Low body temperature **Cerebral small vessel disease burden** ** • Fazekas score** **Markers of systemic inflammation, endothelial function, or thrombosis** ** • Increased MMP-9** • High leukocyte count, fever, high fibrinogen • TNF >14 pg/mL and ICAM-1 >208 pg/mL • Higher ESR • High glutamate • High IL-6 • (diastolic blood viscosity) **Glucose or lipid pathway abnormalities** ** • Diabetes**, glucose (in some, but not all, studies), **HbA1c** • Triglycerides • LDL-cholesterol • Framingham score **Interventions** • Use of statin (reduced risk of END) • Dual antiplatelet therapy (DAPT) (reduced risk of END) • tPA (conflicting data)

*Associated factors are listed in order of our subjective rating of the strength of association based on the number of articles reporting each association in our systematic review. The most consistent associations are shown in* ***bold***.

## Discussion

In our systematic review, we confirmed that END occurs in more than 20% of patients with acute lacunar ischemic stroke and might provide a novel target for clinical trials. A definition of an NIHSS ⩾2 decrease is most used and provides the best between-study homogeneity. In the studies we identified, END is consistently associated with poor functional outcome.

### Associations and mechanisms of END

An understanding of the factors associated with END is important to understand mechanisms and, ultimately, develop novel treatment strategies. In the studies we identified, the rate of END seems to be consistently similar over time, indicating a persistent unmet need for such treatments despite current stroke care (including thrombolysis, mechanical thrombectomy, and stroke unit care). This is perhaps unsurprising since there have been no trials of acute subcortical ischemic stroke due to small vessel occlusion.

#### Features of the acute infarct (morphology, location, size, growth, clinical severity)

Previous studies have investigated associations between acute infarct features (usually defined on DWI) and END. The features associated with END include infarct volume,^34,35^ location in the corona radiata,^36^ posterior locations including the posterolateral striatum,^37–39^ or brainstem,^10^ and irregular infarct shape.^40^ Other studies have described a higher incidence of END associated with proximal infarction (associated with occlusion of the proximal portion of a small perforating artery) as compared to distal infarction (associated with occlusion of the distal portion of a small perforating artery).^41^ The association between infarct size and END may reflect the greater probability of clinical impact with expansion of larger infarcts into eloquent subcortical structures, for example the corticospinal tract.^42^ Alternatively, lesion location may result in some regions being more vulnerable to progression of the infarct itself. For example, progressive occlusion of a perforating artery could lead to enlargement of the area of ischemic injury due to a lack of collateral supply.^43^ Finally, the nature of the initial infarct may be important. Infarcts associated with more edema might cause progressive compression of small adjacent perforators, thus leading to infarct extension and neurological deterioration,^44^ although we are not aware of direct evidence to support this.

#### Presence of BAD

**Figure 3.**
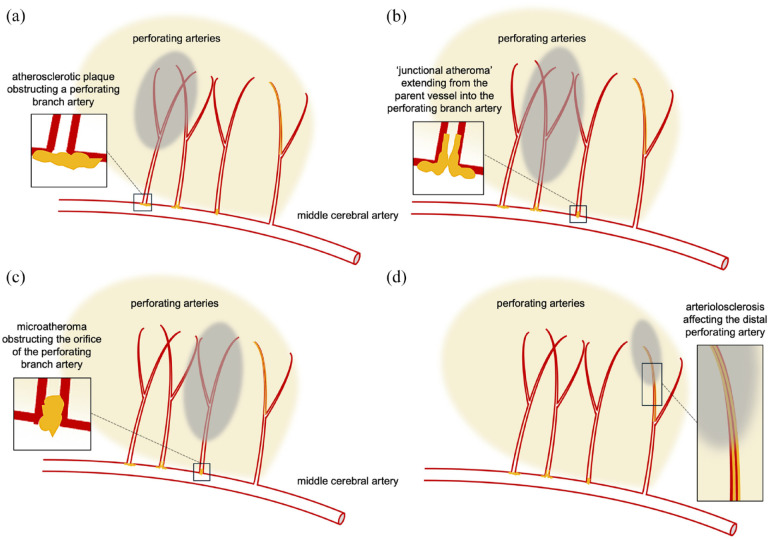
Arterial branch and intrinsic pathology associated with small vessel occlusion: (a) atherosclerotic plaque obstructing a perforating branch artery; (b) “junctional atheroma” extending from the parent vessel into the perforating branch artery; (c) microatheroma obstructing the orifice of the perforating branch artery; and (d) arteriolosclerosis affecting the distal perforating artery branch. The gray areas indicate infarction; branch atheromatous disease (BAD; a to c) typically causes a larger and more proximal area of infarction than occlusion due to arteriolosclerosis.

It is hypothesized that larger, more proximal infarcts in the territory of a perforating small artery are more likely to be related to BAD of the parent artery (Figure 3(a)–(c)), which could in turn be related to END. Postulated mechanisms include propagation of thrombus adherent to an atherosclerotic plaque at the origin of the perforating artery, distal embolization into the perforating artery, or direct occlusion of the perforator orifice by an unstable atheromatous plaque^7,45–47^ Each of these mechanisms could be associated with expansion of the initial DWI lesion, a feature also associated with END. The possible role of an atherothrombotic mechanism in BAD is suggested by a possibly greater efficacy of dual antiplatelet treatment in small subcortical infarction in the CHANCE-2 trial (in a Chinese population where BAD might be expected to predominate over arteriolosclerosis),^48^ but direct evidence for a benefit of dual antiplatelet therapy (DAPT) in BAD remains lacking. In our meta-regression, we found that hypertension, diabetes, and smoking—all risk factors for atheromatous disease—were associated with END.

#### Hemodynamic or autonomic abnormalities

Other important potential mechanisms of END include those related to hypoperfusion, particularly of the at-risk territory, either due to changes in systemic perfusing pressure, impaired cerebral hemodynamics (cerebral perfusion), failure of autonomic control, or a combination of these factors. END, typically within 48–72 h, would be consistent with hemodynamic mechanisms where perfusion in the territory of an occluded small vessel is liable to deteriorate. However, evidence to support the role of reduced perfusion in END after small vessel occlusion is limited. Lacunar infarction and the risk of END are commonly associated with marked hypertension at the time of presentation, but a temporal association with a fall in systemic blood pressure (BP) is less clear; END has, however, been associated with increased variability of BP.^49^ Furthermore, systemically inducing hypertension has been suggested to be beneficial in improving outcomes in small vessel occlusion, implying a potential role for hypoperfusion.^50^

**Figure 4.**
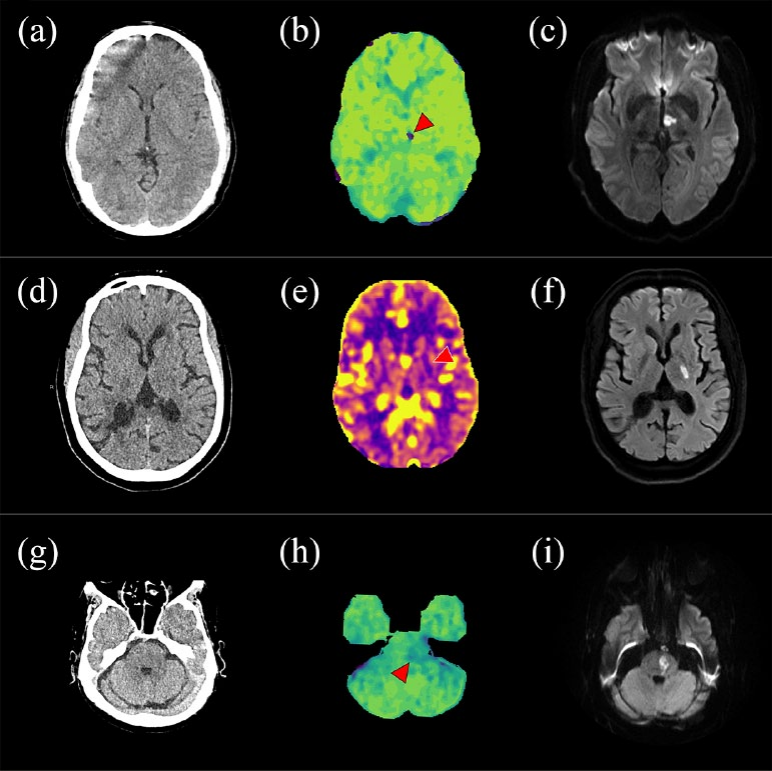
CT perfusion (CTP) in acute lacunar ischemic stroke. A 45-year-old male presented with slurred speech, confusion, and right hemiataxia. Acute ischemia was not identified on the non-contrast CT (NCCT, a) but CTP revealed an area of increased Tmax in the left paramedian thalamus (b, red arrow) that corresponded to diffusion restriction on diffusion-weighted imaging (DWI, c). A 60-year-old male presenting with dysarthria and left-sided facial weakness. An acute infarct was not identified on the NCCT (d), but the CTP cerebral blood flow was lowered in the left internal capsule (e, red arrow), which corresponded to an acute infarct shown on DWI (f). A 70-year-old patient presented with right-sided weakness and dysphagia. NCCT showed a subtle ill-defined region of low attenuation that was indistinguishable from chronic small vessel disease (g). However, CTP showed an area of increased mean transit time in the left hemipons (h, red arrow), which corresponded to an acute infarct on DWI (i).

Focal hypoperfusion on brain imaging has also been associated with END in a limited number of studies. Examples of impaired perfusion in lacunar ischemic stroke are shown in Figure 4.

In a cohort study of 365 patients with acute lacunar stroke, of whom 61 (16.7%) had END, the presence of a hypoperfusion lesion was independently associated with deterioration (odds ratio (OR) = 2.13, p = 0.026).^10^ In one small cohort study,^46^ the authors did MRI and CT perfusion of 26 patients with lacunar infarction within 24 h after onset. Half of the patients (n = 13) deteriorated (END, defined as an increase in NIHSS of ⩾4 points within 7 days from onset). In the END group, subcortical infarctions were enlarged on follow-up MR images. In comparison with a contralateral control region, coronary blood flow (CBF) was lower, and mean transit time (MTT) was higher, in the territory of the affected lenticulostriate artery.

In a small study of 22 patients with lacunar stroke or TIA,^51^ END (defined as an NIHSS worsening of ⩾3 points within 72 h of onset) in four patients (18.2%), all of whom had abnormal perfusion-weighted MRI; however, perfusion-weighted MRI lesions were not associated with infarct growth, nor functional outcome at 90 days. Another study including 43 patients with acute lacunar syndrome and subcortical infarction^32^ used DWI and cerebral perfusion imaging found END (NIHS increased by ⩾2 points within 24 h) in 10 patients. END was predicted when the non-core hypoperfused area overlapped on the corticospinal tract, indicating that infarct location is important, as well as the degree of perfusion reduction. A more recent study included 49 patients with acute subcortical (lacunar) stroke and found that, in multivariate analysis adjusted for covariates, the presence of an increased time to peak (TTP) on CTP was a predictor of END (OR (95% CI) = 4.80 (1.15–20.10), p = 0.03).^52^ Reduced perfusion in the territory of a symptomatic artery and the location of tissue at risk in relation to critical motor tracts (i.e., the corticospinal and associated descending pathways) are likely to contribute to END in acute stroke due to small vessel occlusion. However, the association of perfusion changes with END might be confounded by the association of perfusion abnormalities with larger acute infarcts, which themselves have a higher risk of clinical deterioration. A possible role for hemodynamic mechanisms is also suggested by evidence of impaired cerebrovascular reactivity being more common in patients with END.^53^

Although increasing evidence suggests a critical role for cerebral hypoperfusion in END, previous studies are generally small and potentially subject to selection bias, meaning that further large-scale studies of unselected cohorts including measures of systemic perfusion, cerebral perfusion and infarction, and functional outcome are needed.

#### CSVD burden

Leukoaraiosis (manifest as white matter T2-weighted hyperintensities on MRI or low attenuation on CT) is a common neuroimaging marker of CSVD, mainly arteriolosclerosis. Previous studies^54,55^ have shown relatively consistent associations between leukoaraiosis and the incidence of END but have provided little evidence to understand the pathophysiological relationship. Arteriolosclerosis is associated with abnormal brain parenchymal arterioles, which show a range of structural and functional changes including stenosis, elongation, tortuosity, and impaired autoregulation. As such, an acute occlusion of an arteriolosclerotic vessel may be more vulnerable to progressive occlusion due to a lack of local compensatory mechanisms to limit ischemia. Similarly, adaptive changes in flow through adjacent small vessels after acute small vessel occlusion might be less effective in patients with leukoaraiosis, exacerbating any impaired collateral supply or regional compensatory mechanisms, although direct evidence to support this hypothesis is not currently available. Finally, patients with leukoaraiosis are likely to have reduced functional reserve or compensation throughout their cortical-subcortical networks, making them more vulnerable to clinically eloquent symptoms from the same acute tissue insult, as suggested by a greater incidence of dementia after acute stroke in patients with background leukoaraiosis.^56^

#### Markers of systemic inflammation, endothelial function, or thrombosis

Cerebral ischemic damage is associated with an acute-phase response with an increase in leukocytes, body temperature, and fibrinogen. Clinical studies have confirmed increased levels of proinflammatory cytokines and adhesion molecules in the peripheral blood and cerebrospinal fluid (CSF) of patients with ischemic stroke.^57^ Elevation of inflammatory biomarkers in acute stroke may reflect this inflammatory reaction of the damaged brain tissue itself but could also result from concurrent infection. Prognostic associations have been reported for some inflammatory biomarkers (e.g., interleukin-6 (IL-6) and C-reactive protein (CRP)) in the prediction of functional outcome after stroke,^58,59^ which could be either related to the inflammation itself, but also the degree of tissue injury or intercurrent infection which may each contribute to the inflammatory response. A systematic review which assessed blood markers of coagulation, fibrinolysis, endothelial dysfunction, and inflammation in lacunar stroke versus non-stroke controls and other ischemic stroke subtypes^60^ found that while many markers were higher in lacunar stroke than in non-stroke controls, they were generally lower in lacunar versus non-lacunar stroke. The authors concluded that plasma biomarker elevation in lacunar stroke is likely to reflect the process of having a stroke rather than that systemic inflammation or endothelial dysfunction is specific to lacunar stroke. This interpretation is challenged by data suggesting persisting elevation of biomarkers of endothelial function beyond the acute phase of stroke and associations with progression of white matter hyperintensities in non-stroke populations.^61,62^

Nevertheless, we identified several studies that reported associations between inflammatory biomarkers and END.^30,57,63^ Whether these findings are due to a specific influence of acute-phase inflammatory biomarkers on END or are due to potential confounding effects such as stroke severity (infarct size) or systemic infection remains uncertain. One study noted that the time profile of inflammatory biomarker elevation in END reached a maximum at 8–12 h, consistent with most END occurring at this early time point.^30^ Potential mechanistic hypotheses include cytokines influencing glutamate receptor mediated excitotoxicity, which could contribute to infarcted tissue volume and END^57^ or direct effects of inflammatory processes on the vessel wall, increasing the probability of progressive occlusion.

It has been suggested that END might be due to progressive stepwise thrombosis of branches of small perforating vessels.^35^ Although direct evidence of this is not available, reported associations of END with D-dimer, thrombin, and fibrin formation would be consistent with a progressive stuttering thrombotic process.^64^

#### Glucose or lipid pathway abnormalities

Associations between END and diabetes, glucose, and HbA1c have been noted in some studies. One possible explanation is that these factors are associated with a higher risk of either BAD or more severe arteriolosclerosis, both of which could contribute.

Our study has limitations. To provide a comprehensive overview, we deliberately included all available studies on END; however, this is likely to have contributed to the heterogeneity we observed between studies. We also only included studies published in English, which may have missed some published in other languages and from non-Western settings. In addition, for practical reasons (limited resources), we were not able to undertake a formal risk-of-bias assessment for all included studies.

### Prospects for prevention or treatment of END

No interventions have yet been shown to prevent or reverse END specifically after acute small vessel occlusion in clinical trials. Indeed, we are not aware of any completed, dedicated randomized controlled trials (RCTs) focussing on this question. A recent guideline found no published RCTs directly assessing interventions to reduce END.^6^ Nevertheless, based on observational studies or subgroup analyses from trials, some treatments have been proposed.

#### Antithrombotic agents

Some studies suggest that statins, DAPT, or thrombolysis with tPA might be associated with improved outcomes. In a retrospective study of 458 patients with acute lacunar ischemic stroke, 130 (28%) of patients had END and this was more common in those receiving a single antiplatelet drug (77%) compared with those receiving DAPT (21%).^65^ NIHSS was also better at discharge than admission in the DAPT group (68% vs 35%, p = 0.002), but there was no difference in mRS at discharge (80% vs 73%, p = 0.46). Moreover, in patients with acute lacunar ischemic stroke and evidence of BAD, aspirin plus cilostazol treatment (given within 12 h from symptom) onset was associated with a lower risk of END (18.5% vs 31.4%, p = 0.002) and lower mean mRS at 1 month (1.9 standard deviation (SD) ± 1.5 vs 2.3 SD ± 1.5, p = 0.011).^66^ Intravenous glycoprotein IIb/IIIa inhibitors have also been associated with reduced END in some small observational studies.^67,68^

A pilot multicentre open-label RCT included 343 patients with lacunar stroke and randomized them to cilostazol or no cilostazol, in addition to guideline antiplatelet treatment. In this study, 7/154 (3.2%) allocated cilostazol and 9/175 (6.3%) allocated no cilostazol experienced END (OR = 0.869, 95% CI = 0.304–2.386, p = 0.143).^69^ The Effect of Cilostazol in Acute Lacunar Infarction Based on Pulsatility Index of Transcranial Doppler (ECLIPse) study^70^ evaluated the effect of cilostazol on the change in the pulsatility index (PI) in patients with acute lacunar infarction using serial transcranial Doppler (TCD) examinations. This study reported decreased TCD PIs at 90 days from baseline for the cilostazol group compared with placebo, suggesting pleiotropic effects, such as vasodilation, beyond its antiplatelet activity, but did not report on END.

Another small RCT in a Japanese population included 54 patients with acute lacunar infarcts (n = 29) or BAD (n = 23) within 48 h of stroke and randomized participants to clopidogrel versus no clopidogrel on a background of argatroban and aspirin;^71^ the rate of END was lower in the cilostazol group (0 (0%) versus 4 (16%), p = 0.04) but there was no difference in functional outcome at 3 months.

#### Thrombolysis

There are no dedicated studies of the effect of thrombolysis on the rate of END in acute lacunar ischemic stroke. However, one nonrandomized study in 72 patients with capsular warning syndrome, a condition likely to have a similar pathophysiological basis to END, IV alteplase (versus no IV Alteplase) did not improve the rate of favorable functional outcome (mRS = 0–2) at 3 months (85% vs 84%, p = 0.993).^72^

#### Induced hypertension

Due to the proposed association between hypoperfusion and END, induced hypertension has been proposed as a treatment. Phenylephrine, a sympathomimetic drug which raises BP, has been tested in two small trials (n = 82 and n = 66) of patients with lacunar stroke and END. Patients who received phenylephrine had a lower mean NIHSS at discharge (1.1 SD 1.47 vs 1.86 SD 1.92, p = 0.04; 4.4 ± 2.5 vs 6.0 ± 3.7, p = 0.036) and were more likely to be independent (mRS 0–2) at discharge (62%vs 50%, p = 0.044) or at 3 months (18 (72%) vs 15 (36.6%) p = 0.011).^73,74^ A recent study suggests that earlier induced hypertension is associated with increased odds of neurological improvement, suggesting that the time window may be important.^75^ The PRESSURE (Progressive perforating artery stroke using peripheral dilute norepinephrine; NCT06059144) trial, due to recruiting 358 patients with progressive lacunar ischemic stroke, will investigate the benefit (or harm) of drug-induced hypertension on functional independence at 90 days. The Comparing Between CO2 and Phenylephrine Treatment in Patients With Progressive Lacunar Infarction (CARBOGEN Study, NCT04839224) in Korea plans to compare the effectiveness of carbogen versus phenylephrine in patients with lacunar infarction who suffered neurological worsening.

#### Statins

One study investigated associations of high-intensity statin therapy with END.^76^ Of 492 patients with small cortical infarcts (mean age = 67.2 years, median NIHSS score on admission 3), END occurred in 102 (20.7%). Older age (adjusted odds ratio (aOR) = 1.02; 95% CI = 1.00–1.05; p = 0.017) and branch atheromatous lesion (aOR = 3.49; 95% CI = 2.16–5.74; p < 0.001) were associated with an increased rate of END, while early high-intensity statin therapy (defined as dose expected to reduced low-density lipoprotein cholesterol (LDL-c) by greater than or equal to 50%) was associated with a lower incidence of END than moderate-intensity statin therapy (aOR = 0.44; 95% CI = 0.25–0.77; p = 0.004). In addition, there was a significantly lower incidence of END with early administration (⩽24 h) of high-intensity statins.

#### Neuroprotection

Magnesium is considered a neuroprotective agent with multiple potential actions including inhibition of the neurotoxic effect of excitatory amino acids (particularly glutamate), blockage of calcium entry, and reduction in proinflammatory cytokines, and cell adhesion molecules. In a post hoc analysis of the IMAGES (Intravenous Magnesium Efficacy in Stroke) randomized, double-blind, placebo-controlled trial study, in which magnesium sulfate was given within 12 h of symptom onset in 2386 patients, the overall benefit in clinical outcomes found in patients with noncortical strokes was greatest in patients presenting with lacunar syndromes.^77^

In conclusion, the available observational and trial evidence does not suggest benefit from any specific management strategy (regarding antithrombotic, thrombolytic, or induced hypertensive therapy) to prevent END in acute ischemic lacunar stroke. There is, therefore, an urgent need for targeted trials to investigate rational interventions in acute lacunar ischemic stroke.

### Suggestions for future research directions

This review suggests several potential directions for research to develop effective interventions to reduce END in acute lacunar ischemic stroke.

First, there is a wide range of definitions for END, indicating a need for a standardized definition to allow comparison between studies. Our results suggest that a cutoff of NIHSS change ⩾2 gives a similar incidence to that of NIHSS ⩾1 but with less heterogeneity. A cutoff of NIHSS ⩾4 gave a much lower incidence of END and seems likely to miss clinically important deterioration. Inclusion of a motor component in this score is not necessarily required for identification of END but may be an important predictor of worse clinical outcome and a relevant target in clinical trial design.

Second, trials in acute lacunar ischemic stroke are likely to require early interventions to give the best chance of success. Although END can occur at ⩾72 h, most studies indicate that it is most common in the first 24–48 h. Therefore, trials will need to identify patients with small vessel occlusion as soon as possible after presentation. This can be challenging without hyperacute diffusion-weighted MRI, which is not widely available. One diagnostic strategy is to identify one of the classical lacunar syndromes (pure motor stroke being the most common), but this approach has limited accuracy (sensitivity and specificity reported to be 58% and 45%, respectively),^78^ albeit with some improvement by using an NIHSS ⩽7 cutoff. One study added information supporting a lacunar stroke diagnosis from non-contrast CT (NCCT), reporting improved sensitivity and specificity 83% and 90%, respectively.^79^ In the modern era where CT angiography (CTA) is now becoming routine, future studies should investigate the diagnostic accuracy of a lacunar syndrome, NCCT and CTA (which can quickly exclude a large or medium vessel occlusion with expert interpretation) to identify acute lacunar ischemic stroke for clinical trials. Where CTP is available, focal hypoperfusion may also improve accuracy of diagnosis (Figure 3) but hypoperfusion is only present in a small subset of patients with small subcortical infarcts, yielding limited diagnostic accuracy (one recent systematic review found a sensitivity of CTP ranging from 0% to 62.5% and specificity from 20% to 100%).^80^

Third, the pathophysiology of vessel dysfunction or occlusion is different in acute lacunar ischemic stroke associated with BAD compared with intrinsic arteriolosclerosis. Although infarct diameter has been used to infer a branch atheromatous cause (on the basis that such disease will cause a larger area of infarction), it is desirable to directly identify such atheroma in the acute phase; recently, developed gadolinium-enhanced vessel wall imaging has a promising role in this regard.^81^ Effective therapies in BAD may target distinct mechanisms of END, for example athero-thromboembolism.

Fourth, given recognized risk factors for END, development of simple tools to identify patients at an increased risk of END may be important to enrich populations for future clinical trials and identify patients most likely to benefit from treatment.

Finally, definitive evidence that a mechanism is responsible for END after acute lacunar ischemic stroke will require testing of specific interventions in dedicated randomized controlled clinical trials and potentially provide evidence for interventions targeting specific mechanisms with the potential to improve clinical outcomes. For example, embolism into a perforator form BAD might be reduced by interventions aiming to reduce this (e.g., DAPT); indeed, subgroup analyses of previous clinical trials may provide further insights into the role of thrombotic mechanisms, for example recent trials comparing thrombolysis and DAPT in minor stroke.^82^ Alternatively, reduced perfusion might rationally be treated by strategies to improve cerebral blood flow through a symptomatic artery; for example, ongoing studies testing induced in END associated with small vessel occlusion. Even with limited knowledge of predictors and mechanisms, END itself may still provide a useful outcome measure with which to select potential interventions specific to acute lacunar ischemic stroke.

## Supplemental Material

sj-docx-1-wso-10.1177_17474930241273685 – Supplemental material for Early neurological deterioration in acute lacunar ischemic stroke: Systematic review of incidence, mechanisms, and prospects for treatment Supplemental material, sj-docx-1-wso-10.1177_17474930241273685 for Early neurological deterioration in acute lacunar ischemic stroke: Systematic review of incidence, mechanisms, and prospects for treatment by David J Werring, Hatice Ozkan, Fergus Doubal, Jesse Dawson, Nick Freemantle, Ahamad Hassan, Suong Thi Ngoc Le, Dermot Mallon, Rom Mendel, Hugh S Markus, Jatinder S Minhas and Alastair J S Webb in International Journal of Stroke
