# Supplementary material for: Early neurological deterioration in acute lacunar ischemic stroke: Systematic review of incidence, mechanisms, and prospects for treatment
Source: Int J Stroke. 2024 Sep 5;20(1):7–20. doi: 10.1177/17474930241273685 (PMC11669265; doi:10.1177/17474930241273685)
Supplement: sj-docx-1-wso-10.1177_17474930241273685 – Supplemental material for Early neurological deterioration in acute lacunar ischemic stroke: Systematic review of incidence, mechanisms, and prospects for treatment [file sj-docx-1-wso-10.1177_17474930241273685.docx]

**Supplementary material for: Early neurological deterioration in acute lacunar ischaemic stroke: incidence, mechanisms, and prospects for treatment**

**Supplementary Figure 1.** *Publication bias funnel plot*


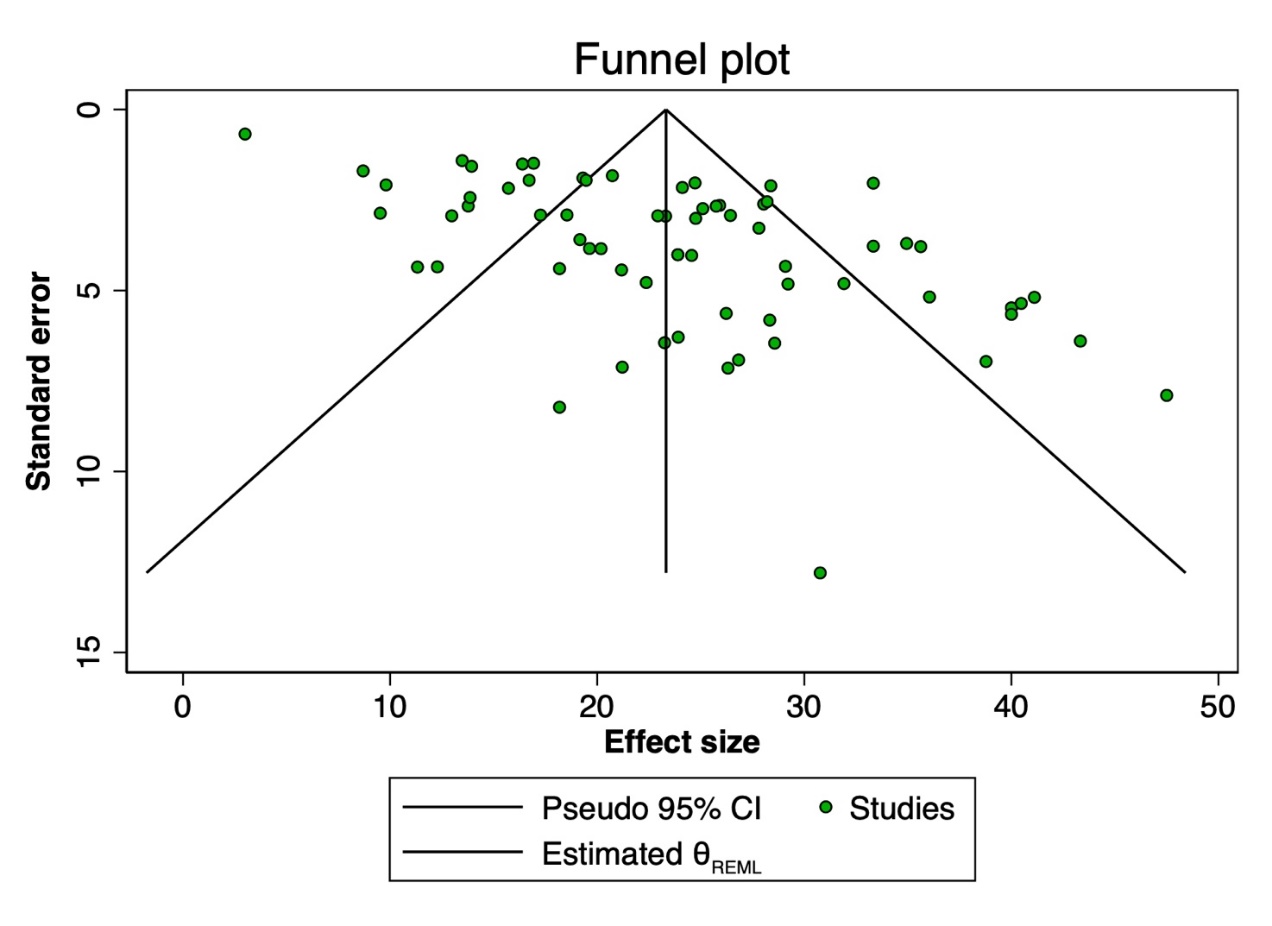


*****Funnel plot the triangle line represents the region where 95% of the data points suggest studies absence of publication bias. Whereas those outside the line suggests presence of publication bias.

**Supplementary Table 1.** *Populations, definitions, incidence, timing, and associations of early neurological deterioration in acute subcortical (lacunar) ischaemic stroke*

| **Study Author, year** | **Population included** | **Definition of END** | **Rate (incidence) of END** | **Timing of END, if reported** | **Factors associated with END** | **Was END associated with worse functional outcome?** |
| --- | --- | --- | --- | --- | --- | --- |
| Audebert, 2004 | N=46, clinical lacunar stroke, <2cm on MRI or CT | Worsening by ≥1 point on the NIHSS in motor function, measured at ≤12h, day 1, day 2 and hospital discharge | 11/46 (24%) | (9/11 in the first 24h) | High leucocyte count, fever, high fibrinogen, glucose | Yes. Barthel index at 90 days worse in those with END |
| Bashir, 2022 | N=187 consecutive patients presenting with one of the five classic lacunar syndromes (LS) and absence of vascular occlusion, perfusion deficit or symptomatic stenosis. No lesion on MRI or CT >20mm. | Deterioration of 4 total NIHSS points, or 2 NIHSS points for limb paresis within one week. | 52/187 (27.8%) | NR | History of diabetes mellitus, higher diastolic blood pressure at admission, clinical deficits consistent with a pure motor syndrome and asymptomatic intracranial atheromatosis or stenosis in non-symptomatic territory were independent predictors | Yes. Good outcome (mRS 0-2) 44.4% for END vs 86.1% at 90 days |
| Berberich, 2019 | N=458, lacunar stroke symptoms and detected lacunar infarction by magnetic resonance imaging, diameter ≤20mm | Deterioration of ≥3 total NIHSS points, or ≥2 NIHSS points for limb paresis or description of fluctuating clinical symptoms which might not be well reflected by the NIHSS in electronic medical reports (within 5 days) | 130/458 (28%) | NR | Male gender, motor paresis, internal capsule or basal ganglia infarction, “speckled DWI lesion, Fazekas score. | Yes |
| Castellanos, 2002 | N=113  consecutive patients with lacunar infarction included within the first  24 hours of the onset of symptoms | Fall of ≥1 points in the motor items of Canadian Stroke Scale between inclusion and 48 hours | 27/113 (23.9%) | NR | TNF >14  pg/mL and ICAM-1 >208 pg/mL were independently associated with END and poor functional outcome | Yes. 48% poor outcome (death or Barthel Index <85 at 3 months) with END vs 15% without END |
| Chen, 2017 | N= 687 consecutive patients with acute ischemic stroke and also with admission NIHSS score below 12 points | increment of NIHSS score ≥2 points in the first 72 h after admission | 63/251 (25.1%) | NR | No association of SVD burden with END. | NR |
| Cho, 2009 | N=77 patients with unilateral small subcortical infarction (SSI) on diffusion-weighted MRI (DWI) ≤2cm diameter within 48 h after stroke onset. Did not exclude other possible mechanisms. | Increase of the National Institutes of Health Stroke  Scale (NIHSS) score by ≥2 during the first week | 14/77 (18.1%) | NR | Increase in lesion volume associated with END | Yes. Good outcome (mRS 0-2) at 1 month 41.2% in those without END and 11.7% in those with END |
| Duan, 2015a | N=312, (Jinling hospital); patients with acute SSSI (single small subcortical infarct) in the perforator territory of MCA | Increase in NIHSS score ≥2 points during the first 72 h after admission | 88/312 (28.2%) | NR | Proximal SSSI pattern in patients with large artery atherosclerosis or SVD, but not patients with cardioembolism  Female sex | NR |
| Duan, 2015b | N=227, (Yangzhou  Hospital); acute  SSSI | Change in National Institutes of Health Stroke Scale score ≥2 points in the first 72 h after admission | 60/227 (26.4%) | NR | Proximal SSSI pattern  Female sex | Yes. Of those with poor outcome (mRS≥3) 47.8% had END, compared to 19% in those with good outcome |
| Duan, 2017 | N=296 (Yangzhou 2013-2016), acute SSSI with or without parent artery disease (PAD) | NIHSS score increased ≥2 during the first 72 h | Overall, 83/296 (28%) | NR | Higher rate of END in those with PAD (38.5% vs 24.3%). BP parameters were not associated with END or functional outcome | Yes. Of those with a poor outcome, 38/54(70.4%) had END, compared to 30/242(12.4%) of those with a good outcome (mRS 0-2 at 90 days) |
| Farooqui, 2022 | N=49, DWI+ (≤20mm diameter) and SVD mechanism as classified by TOAST, with CTP available | ≥1‑point worsening on a modified National Institutes  of Health Stroke Scale (NIHSS) | 19/49 (38.8%) | NR | Increased TTP on CT perfusion associated with END. Age, gender, race, decrease in SBP or DBP, modified NIHSS score, and the stroke lesion size were not associated with END. More patients in the ND group were made to have head of bed flat (5 [26%] vs. 1 [3%], P = 0.03). | More patients with ND were discharged to inpatient rehabilitation compared to those who did not have ND (53% vs 20%), but it was not statistically significant (p=0.1) |
| Feng, 2013 | N=204, DWI <20mm on T1, SVD mechanism as classified by TOAST | Increased NIHSS score on day 14 | 20/204 (9.8%) | NR | Irregular infarct shape (but unclear how this classification worked) | NR. Irregular infarction associated with mRS at 3 months |
| Feng, 2014 | N=435, single acute subcortical infarction (<1.5 cm in diameter) | Worsening by 2 points or more in the National Institutes Health Stroke Scale  (NIHSS) score, or by 1 point or more in the NIHSS score for motor function within  1 week after stroke onset | 84/435 (19.31%) | NR | Severity of leukoaraiosis, baseline NIHSS  score, and diabetes were independently associated with ND | NR |
| Forti, 2023 | N=834, classical lacunar syndrome with infarct <15mm on CT or MRI | Increase in NIHSS score ≥4 points or death within 24 h after admission | 19/634 (2.3%) | NR | Serum glucose measures not associated with deterioration in lacunar stroke | NR |
| Gökçal, 2017 | N=120 with Isolated pontine infarction on DWI. | Increase in the National Institutes of Health Stroke scale ≥2 units within 5 days after onset | 23/120 (19.2%) | NR | Basilar artery branch disease more common in those with END (65.2% vs 36.1%) | Yes. 40.5% of patients with PD and 9.6% of patients without PD had poor functional outcome at 3 months (p <0.001) |
| Griebe, 2014 | N=537, supratentorial, DWI+, ASCO classified SVD without competing mechanism, DWI lesion ≤20mm | Increase in NIHSS between admission and day 5 | 179/537 (33.3%) | NR | Not reported as the focus was on thrombolysis and functional outcome | NR |
| Ha, 2018 | N=60, acute lacunar infarction defined as a single lesion caused by occlusion of deep perforators  sized less than 30 mm in largest diameter with high signal intensity on DWI and low signal intensity on ADC images at subcortical  deep brain structures, including basal ganglia, corona radiata, thalamus, and brainstem; excluded competing mechanisms. Autonomic function tests were performed. | Increase in National Institute of Health Stroke Scale (NIHSS) over 1 point during hospitalization without recurrence of new brain lesion | 17/60 (28.3%) | Within 24h in 15 of 17 patients | BP response in Valsalva test (measure of sympathetic function) | Yes. END in 15 (53.6%) of patients with poor outcome compared with 2 (6.3%) patients with favourable outcome (mRS≤1) |
| Hallevi, 2012 | N=202, acute subcortical ischemic stroke (focal deficit lacking ‘cortical signs’ (such as hemianopsia or aphasia) | Increase of ≥2 points on the NIHSS (or 1 point on the  motor subcomponent) from the baseline exam (appears to have been recorded at any time during admission) | 28/202 (14%) | at a median of 2 days after stroke onset | Intracranial atherosclerosis affecting the appropriate arterial territory | NR |
| Huang, 2014 | N=43, acute lacunar infarct and classic lacunar syndrome | NIHSS increase ≥2 within 72 hours | 10/43 (23.2%) | 21.5+/- 15.7 hrs. | END was predicted when the non-core hypoperfused area overlapped on the corticospinal tract | NR |
| Hwang, 2008 | N=76, OCSP lacunar syndrome and “clinically relevant” appropriate infarct on DWI | ≥1 point in the motor sub-items of the NIHSS score or 2 points in other NIHSS sub-items during the first  72 h after symptom onset | 17/76 (22%) | NR | tPA appeared to increase END in those classified as small vessel occlusion | No association between END and functional outcome |
| Im, 2021 | N=268, subcortical infarctions with diabetes or prediabetes | Increase of motor score of ≥ 1 on the upper or lower limb items of NIHSS during the first 72 h after stroke onset | 69/268 (25.7%) | NR | Increased HbA1c associated with progressive motor deficit | NR |
| Jang, 2020 | N=205, single subcortical infarction (SSI) without any relevant artery stenosis, <24h from symptom onset | Worsening by 2 points  in the total NIHSS score or 1 point in the motor items of  the NIHSS score compared to the initial NIHSS score during  the first week of admission or before hospital discharge. | 47/205 (23%) | NR | Length of infarcted lesion (three or more slices showing the cerebral infarction on a transverse plane) | Yes. END was associated with increased risk of poor outcome (mRS 1.54 vs 0.41). |
| Jang, 2023 | N=492 patients with single small subcortical infarction (SSSI) who received statin therapy within 72 h of symptom onset from a prospective stroke registry | Any new neurologic symptoms/signs or neurologic worsening satisfying one or more of the following criteria: (1) an increase of 2 points in total NIHSS score, (2) an increase of1 point in the motor NIHSS score, and/or (3) an increase of 1 point in the consciousness  Score | 102/492 (20.7%) | Median time from onset to END was 39.0 (23.0–77.0) h | Early high-intensity statin therapy was associated with a lower incidence of END | NR |
| Jeong, 2015 | N=586, single ischemic lesions with an axial diameter ≤20 mm with a patent adjacent relevant artery (degree of stenosis ≤50%) and without atrial fibrillation | (1) an increment in the total NIHSS score of ≥2 points, (2) an increment in the consciousness score (1a–1c) of ≥1, (3) an increment in the motor score (5a– 6b) of ≥1, or (4) any new neurological deficit (even unmeasurable by NIHSS scores) within 3 weeks | 79/586 (13.5%) | Time intervals from admission to END and from onset to END were 30.0±32.6 and 40.3±36.0 hours, respectively | Patients with relevant artery stenosis (adjusted odds ratio, 1.91; 95% confidence interval, 1.13–3.21) and branch atheromatous lesions (adjusted odds ratio, 2.98; 95% confidence interval, 1.80–4.93) had significantly higher odds of END | Yes. Patients with END showed higher frequencies of modified Rankin Scale scores of 3 to 6 after 3 months compared with patients without END (49% versus 23%) |
| Jiang, 2019 | N=160, single small subcortical infarct (2.0 cm in diameter). BAD or cardioembolic sources excluded | Worsening by ≥1 point in National Institutes Health Stroke Scale (NIHSS) motor score within 72 hr from onset.  17 (29.8%) patients showed 1‐point worsening in NIHSS score, 25 (43.9%) pa‐ tients experienced 2‐point worsening, 15 (26.3%) ones underwent an increase by 3 points, while none of them had >=4 points worsening in our study | 57/ 160 (35.6%) | NR | Severe WMHs  (OR = 4.892; 95% CI = 2.011–11.904, p = 0.016), moderate‐ and high‐grade basal ganglia EPVS (OR = 2.970; 95% CI = 1.861–6.121, p = 0.009), and total cSVD score (OR = 3.359; 95% CI = 2.016–5.599, p = 0.010) were associated with progression | NR |
| Jin, 2023 | N=280, acute single small subcortical infarction (SSSI) with antiplatelet therapy without carotid artery stenosis. | Increment in the National  Institutes of Health Stroke Scale (NIHSS) score of ≥ 2 points or any new neurological deficit within 48h and lasting ≥24h | 44/280 (15.7%) | END occurred in 28 (63.6%) patients during sleep, and END occurred in 16 (36.4%) patients during wakefulness or activity | History of hypertension, infarction in internal capsule or a higher level of LDL-C  Two-thirds occurred during sleep | Yes. END associated with increased risk of poor 3-month outcome (aOR 5.74). |
| Kalowska, 2018 | N=13, “lacunar infarcts” | Permanent decrease of 6 3  Scandinavian Stroke Scale (SSS) points for speech or 6 2 SSS points for consciousness or 6 2 SSS points for limb strength, when assessed at baseline compared to the day after admission and daily during the following week | 4/13 (31%) | 11/15 (73.3%) patients within 24 h from symptom onset and 4 patients between 24 h and 7 days | Larger DWI volume >1.5cm3 | NR |
| Kim, 2006 | N=49 patients with acute lacunar infarction (as defined by clinical and MRI criteria within 48 h after stroke onset) | Increased NIHSS motor subscore more than 1  point higher than the initial score | 14/49 (28.6%) | 6 (42.9%) showed neurological worsening within 24 h of initial stroke symptoms, 5 (35.7%) within 48 h, 2 within 72 h and 1 patient after 72h | Increased MMP-9 levels and higher ESR in those who progressed | NR |
| Kim, 2008 | 167 consecutive patients with deep subcortical infarction  in the anterior circulation, within 24h. No upper size cutoff. | modified National Institutes  of Health Stroke Scale (mNIHSS) motor score of ≥1 during the first week of symptom onset | 23/167 (13.8%) | NR | involvement of the posterolateral striatum, high blood pressure |  |
| Kim, 2015 | N=274, single subcortical infarction (SSI) | Increase of >1 points in the motor portion of  the National Institute of Health Stroke Scale (NIHSS) within 72 h from MRI acquisition | 71/274 (25.9%) | NR | Diffusion < perfusion mismatch, NIHSS on admission | NR |
| Koh, 2011 | N=206, first lacunar stroke, DWI maximum diameter 15mm | Increase in more than two points on the National Institutes of Health Stroke Scale Score baseline  14 days after the onset of lacunar stroke | 48/206 (23.3%) | NR | Existence of microbleeds (RR = 2.47, 95% CI = 1.25–3.83) and increased free, active MMP-9 (RR = 1.10 per 10 ng/ml, 95% CI = 1.03–1.19) were identified as independent risk factors for neurological deterioration after adjusting for  potential confounders | NR |
| Kwon et al, 2011 | patients with acute lacunar infarct within 48 h after  symptom onset, lesion diameter <1.5cm | An increment of the National Institutes of Health Stroke Scale (NIHSS), ≥1 in motor  power, or ≥2 in any scores during the first week | 17/131 (13%) | 52.9% of the END cases occurred within the first 48h after admission | Highest quartile of triglycerides. | NR |
| Lee, 2017 | N=89 with lacunar infarction (also 80 with striatocapsular infarction, >3m diameter) | Worsening of two or more points on the NIHSS or one or more point on the motor scale during admission | 26/89 (29.2%) | NR | HbA1c, diabetes, NIHSS, MCA pulsatility index higher in people with lacunar stroke who had END | NR |
| Lee, 2022 | N=178 with lacunar infarction who were admitted within 72h. TOAST classification with infarct <20mm on DWI. | Increase in the National Institute of Health Stroke Scale (NIHSS) score ≥2 within 24h of admission | 33/178 (18.5%) | NR | Diastolic blood viscosity associated with END | NR |
| Lin, 2022 | N= 86 patients with an anterior circulation subcortical infarction within 48h after onset | Increase of ≥ one point in motor power or ≥ two points in the total National Institute of Health Stroke Scale score within 7 days after admission | 31/86 (36%) | NR | Infarct slice number ≥ three slices proved to be independently associated with END | NR |
| Moon, 2008 | N=80 consecutive patients with striatocapsular-region infarction, as defined  by clinical and MRI criteria, within 24 hours after stroke onset. No upper size limit on DWI. | An increase of at least 2 points in the motor item of the NIHSS lasting for at least 24 hours and within 7 days of stroke onset. | 32/80 (40%) | NR | history of hypertension and an initial infarct extent of ≥15 mm | NR |
| Naess, 2018 | N=114 with lacunar infarction | At least one score with increased NIHSS score ≥3 points compared  with NIHSS score on admission | 28/114 (25%) | Most appear to occur in the first 12 hours (Fig 1). | Low body temperature, high systolic blood pressure, and short time from onset to admission | NR |
| Nagakane, 2008 | N=61 consecutive patients with a supratentorial  lacunar infarct, who were admitted within 48 h | The progressive-type stroke  (PS) was defined as deterioration in motor  deficits within 7 days after stroke onset from mild to either moderate or severe, or from moderate to severe. Deficits were categorized into three degrees: mild (grade 0 or 1 NIHSS motor rating), moderate (grade 2), or severe (grade 3 or 4). | 16/61 (26%) | NR | Fluctuating or progressing onset, leg-predominant  motor deficits on admission and corona radiata lesion on diffusion-weighted MRI | Yes. Patients in the PS group had poorer outcomes than those in the non-PS group. Both the modified Rankin Scale (range, 1–4 vs. 0–3; median, 4 vs. 1) and the Bar- thel Index (range, 20–100 vs. 45–100; median, 60 vs. 100) at discharge were significantly different between the two groups (p <0.001, p<0.001, respectively). |
| Nakamura, 2022 | N=84 patients with acute infarction in the lenticulostriate artery (LSA) territory | Worsening of the pre-symptomatic modified Ranking Scale (mRS) by  two or more stages at discharge | 34/84 (40.1%) | NR | Framingham Risk Score  (FRS) and Suita score (SS), total and LDL-cholesterol | NR |
| Nakase, 2013 | N=57 Lacunar infarction defined as an intracerebral lesion <15 mm in diameter and fewer than 3 slices or a lesion within the pontine parenchyma | >2-point increase in the National Institutes of Health Stroke Scale within 48 hours of stroke onset | 7/57 (12.3%) | NR | Higher rate of progression in Branch Artery Disease (BAD); 38.1% | NR |
| Nakase, 2014a | N=453 total with deep penetrating arterial infarction. N=233 treated with cilostazol, N=220 treated with standard care | Increase in the NIHSS score more than 2 points within 48 hours from the onset | Overall 112/453 (24.7%)  69/220 (31.4%) (standard care)  43/233 (18.5%) (cilostazol) | NR | Branch Artery Disease (BAD) associated with progression; cilostazol associated with non-progression | NR |
| Nakase, 2014b | N=38 acute pontine infarctions were confirmed by magnetic resonance imaging | NIH Stroke Scale ≥2 increase within 72 h | 10/38 (26.3%) | NR | END related to the severity of basilar artery atherosclerosis. | Yes. END patients showed severer neurological deficits indicated by NIHSS at 1 month compared with non-END (P = 0.05: 5.9 and 3.6, respectively) |
| Nakase, 2015 | N=166 in total; N=124 lenticulostriate; N=42 pontine perforator | Score on the National Institutes of Health  Stroke Scale increased more than 1 point within 7 days  after the onset of medical treatments | Overall 58/166 (34.9%)  44/124 lenticulostriate (35.5%)  14/42 pontine perforator (33.3%) | NR | Higher rate of progression with Branch Artery Disease (BAD) (45.5%) than lacunar infarction (18.5%).  Percentage of patients with neurologic  worsening was significantly higher in the cases with  lesion enlargement compared with those with stable lesions | NR |
| Nam, 2023 | N=604 consecutive patients with SSI who underwent MRI within 24h. | Increase of ≥ 2 in the total National Institutes of Health Stroke Scale (NIHSS) score or ≥ 1 in the motor NIHSS score | 99/604 (16.4%) | NR | Infarct growth volume associated with END overall, and in the subgroup with distal but not proximal SSI. | NR |
| Nannoni, 2015 | N=94, lacunar syndrome with a subcortical infarct including AChA and striatocapsular infarcts. | Deterioration of NIHSS motor score of ≥1 points during the first 72 h after stroke onset or fluctuation of symptoms (neurological worsening alternating with improvement with at least some residual deficit) | 30/94 (31.9%)  36% rate of progression in the group with pure motor syndrome (14/45) or sensorimotor syndrome (12/ 27). None of the three patients with pure sensitive syndrome underwent neurological progression. | NR | Infarct diameter ≥15 mm and severe leukoaraiosis independently associated with END (OR = 6.3, 95% CI 2.0–19.6 and OR = 5.9, 95% CI 1.3–25.7, respectively) | NR |
| Ohara, 2010 | N=60 consecutive patients with acute lacunar  infarction in the lenticulostriate artery territory within 24 h after onset. | Deterioration of National  Institutes of Health Stroke Scale (NIHSS) motor score ≥1 during the first 7 days after admission | 26/60 (43.3%) | NR | “posterior corona radiata type” (more than half of the DWI corona radiata lesion adjacent to the lateral ventricle in the posterior region) independently associated with progression | NR |
| Ois, 2008 | Total N=1093, of whom 276 were classified as “small vessel” stroke (TOAST classification)  Eleven patients had ACA infarcts, 900 MCA territory infarcts, 51 PCA infarcts, and 131 infratentorial VB infarcts | ≥4 points increase in the NIHSS within 72h | 24/276 (8.7%) | NR | Not reported for the small vessel subgroup | NR |
| Pan, 2023 | N=107, small subcortical infarction in penetrating artery territories were recruited, with perfusion MRI within 24h of stroke onset. | Elevation of 2 points on the NIHSS within 72 h of stroke onset | 21/107 (19.6%) | NR | Perfusion defects were associated with a higher rate of END (34.5%vs. 3.8%; p < 0.001); adjusted analyses of END predictors not reported | NR for END, but perfusion deficits were associated with worse functional outcome at 3 months |
| Poppe, 2009 | N=22, lacunar stroke or TIA were selected from a prospective MR imaging study | NIHSS worsening of ≥3 points within 72 h of event | 4/22 (18.2%) | NR | Abnormal perfusion-weighted imaging | NR |
| Ryu, 2012 | N=105, confirmed “lacunae-sized acute ischemic infarcts” in the penetrating arterial territories on diffusion-weighted MRI. Based on  diffusion-weighted MRI, the shape of ischemic infarcts was divided into oval or ‘conglomerated  beads’ shapes | Any increase of NIHSS score at the time of discharge | 10/105 (9.5%) | NR | Early neurologic deterioration was more commonly observed in the conglomerated beads shape  group than in the oval shape group (7 [20.6%] vs 3  [4.2%], p=0.012). | NR |
| Saji, 2012 | N= 156 consecutive first-  Ever ischemic stroke patients with acute deep subcortical infarction | Increase of ≥2 points in the National Institutes of Health Stroke Scale score or increase of ≥1 point in limb weakness in the National Institutes of Health Stroke Scale score during the 7 days after stroke onset | 52/156 (33.3%) | The average time to detect PND from the onset of ischemic stroke was 24 hours (interquartile range, 17–30 hours) | Arterial stiffness (baPWV) (≥18.24 m/s; odds ratio, 8.22; 95% confidence interval, 2.55–31.9), large infarct  size (≥15 mm; odds ratio, 2.76; 95% confidence interval, 1.01–7.92), and ≥3 infarct slices on serial axial diffusion-weighted  imaging (odds ratio, 3.38; 95% confidence interval, 1.22–10.0) were associated with deterioration. | NR |
| Saji 2018 | N=41 patients with stroke  due to recent small subcortical infarcts within 48 hours of onset | An increase of greater than or  equal to 2 points in the NIHSS score during the 3 days  after stroke onset | 11/41 (26.8%) | NR | age (OR 1.14, 95%CI 1.03-1.31, P = .008), diabetes mellitus (OR 14.0, 95%CI 1.67-225.9, P = .013), dyslipidemia  (OR .1, 95%CI .002-0.92, P = .042), and NIHSS score on admission (OR 2.06, 95%CI 1.34-4.22, P < .001) were independently  Associated with END | Yes. END was associated with lower rate of good outcome mRS 0-2) at 3 months (18.2% vs 93.3% for those without END) |
| Sakamoto, 2012 | Total N=147, of whom N=53 had small vessel occlusion (TOAST criteria) | ≥2 increase in the NIHSS score during 7 days from admission | 6/53 (11.3%) | NR | Augmentation index (measure of arterial stiffness) associated with progression. | NR |
| Serena, 2001 | N= 113 consecutive patients with lacunar infarct, defined by clinical and computed tomography/  magnetic resonance imaging criteria, within the first 24 hours after stroke onset | Decrease of ≥1 points in the motor items of the Canadian Stroke Scale in the first 48 hours after admission | 27/113 (23.9%) | NR | High glutamate/ low GABA in serum associated with progression.  Blood glucose at 24 and 48h higher in those with END | NR |
| Shin, 2017 | N= 109 consecutive patients with SSI examined by whole supratentorial brain CTP and follow-up DWI.  Branch atheromatous disease (BAD) in N=32  BAD-type perfusion  pattern when (1) the uninterrupted hypoperfused area (TTD ≥ 5 seconds) that was connected to the inferior portion of the “core” ischemic lesion (indicated by CBV <1.2 mL/ 100 mL) was located less than 5mm from the cerebrospinal fluid (CSF) perforators interface on both coronal and sagittal reformatted TTD maps | an increase by at least one in the NIHSS motor score (range 0-8) of the affected upper and lower limbs after the DWI acquisition and within 5 days from stroke onset, as witnessed by 2 independently trained NIHSS neurologists and stroke nurses | 22/109 (20.2%) overall  For BAD 13/32 (40.6%)  Without BAD 9/77 (11.7%) | NR | Progression more frequent in BAD perfusion pattern (40.6% vs 11.7%) | NR |
| Siegler, 2016 | N=1,387 DWI confirmed acute ischaemic stroke of whom 206 had END. | Worsening of 2 or more points on the National Institutes of Health Stroke Scale during a 24-hour period of hospitalization | 51/206 (24.8%) | NR | NR for small vessel occlusion | NR |
| Takase, 2011 | N=40 acute lacunar infarction  in the lenticulostriate artery (LSA) territory | Deterioration of NIHSS motor score of ≥2 points during the first 7 days after admission | 19/40 (47.5%) | NR | DWI area was significantly larger in those with END, 0.98cm^3^ was the optimal cut-off | NR |
| Takeuchi, 2016 | N=488, consecutive patients with acute ischemic stroke  in penetrating artery territories with less than 50% stenosis in parent vessels | Any increase by 1 point  or higher in the National Institutes of Health Stroke Scale score | 68/488 (14%) | 23% of these patients had END within the first 24 hours and 44% within the subsequent  24 hours after admission | Initial high blood pressure, diabetes, and low BMI on  admission were associated with early progression | Yes. mRS at 3 months was higher in those with END (3(2-4) vs 1(0-2), p<0.0001, though END was not associated with functional improvement. |
| Tan, 2022 | N=638 consecutive acute lacunar stroke patients defined by magnetic resonance imaging | Persisting increase in National Institutes of  Health Stroke Scale (NIHSS) score of ≥ 2 points post admission | 108/638 (16.9%) | 67.6% (73/108) of the END  cases occurred within 24 h after admission, and 94.4% (102/108) occurred within 72 h after admission | Admission NIHSS score (adjusted odds ratio (aOR) 1.132, 95% confidence interval (CI) 1.046–1.225, p = 0.002), female  (aOR 2.752, 95% CI 1.277–5.933, p = 0.010), admission systolic blood pressure (SBP) (160–179 mm Hg) (aOR 9.395, 95%  CI 4.310–20.479, p < 0.001) and admission SBP (⩾180 mm Hg) (aOR 16.030, 95% CI 5.991–42.891, p < 0.001) were significantly associated with END | Yes. END (aOR 12.374, 95% CI 6.881–22.254,  p < 0.001) and higher admission NIHSS score (aOR 1.488,  95% CI 1.359–1.629, p < 0.001) predicted unfavorable outcome  at discharge (mRS>2) |
| Umemura, 2014 | N=85 consecutive patients with acute small subcortical infarcts <20mm diameter in the lenticulostriate  artery territory within 24 hours of symptom onset who had serial DWI; atrial fibrillation and large artery disease excluded | Increase of ≥2 points in the National Institutes of Health Stroke Scale score during the first 5 days after admission | 18/85 (21.2%) | NR | Albuminuria was  associated with END after adjustment for age, low estimated glomerular filtration rate (<60 mL/min per 1.73 m2),  and infarct volume on initial DWI (odds ratio, 6.64; 95% confidence interval, 1.62–27.21; P=0.009) | NR |
| Vahidy, 2014 | N=90, patients presenting within 24 hours of symptom onset with clinical and radiologic (CT or MRI) evidence of subcortical stroke. No upper size limit. | Motor score increase of at least 1 on the NIH Stroke Scale | 37/90 (41%) | Median (IQR) time to deterioration from was 21 (5.2–41) hours from stroke onset. 75.6% of patients (28/37) deteriorated within 24 hours from the time of the first NIHSS | Administration of tissue plasminogen activator was significantly associated with deterioration (hazard ratio 2.25; 95% confidence interval [CI]: 1.13–4.49) | Yes. Deterioration conferred an increased risk of poor outcome (modified Rankin Scale scores 3–6) at discharge (relative risk: 1.80; 95% CI: 1.71–1.93) |
| Vila, 2000 | N=231, of whom 33 had small vessel mechanism (TOAST classification) | Canadian Stroke Scale (CSS)  score fall of at least 1 point during the first 48 hours after admission | 7/33 (21.2%) | Eighty-three patients (35.9%) worsened within the first 48 hours after stroke onset: 41 (17.7%) worsened by 1 point, 24 (10.4%) by 2 points, and 18 (7.8%) by ≥3 points | IL-6 levels on  admission associated with early clinical deterioration, without regard to the initial size, topography, or mechanism of the ischemic infarction | NR |
| Vynckier, 2021 | N=365 consecutive patients with MRI-defined lacunar stroke who presented within 12 hours after symptom onset from a prospective stroke database | Any persisting increase in NIHSS score of ≥2 in the  first 24 hours | 61/365 (16.7%) | NR | Lower NIHSS score on admission (per point, aOR 0.81, p = 0.006), capsular warning syndrome (aOR 7.00, p < 0.001), ventral  pontine infarct (aOR 3.49, p = 0.008), and hypoperfusion lesion on imaging (aOR 2.13, p =0.026) were associated with END. Acute dual antiplatelet therapy was associated with reduced  risk of END (aOR 0.10, p = 0.04) | Yes. END was asso- ciated with a less favorable outcome at 90 days (aOR 0.13 [95% CI 0.05–0.30] p < 0.001) for good functional outcome (Modified Rankin Scale Score 0–2) at 90 days |
| Wei, 2022 | N=168, consecutive patients with acute small subcortical infarct treated with IVT | ≥2-point increase of NIHSS from baseline to 24 hours, “without straightforward causes” | 29/168 (17.2%) | NR  Note: Among the patients with END, five patients had symptomatic intracranial hemorrhage, nine patients early recurrent stroke, and one patient post- stroke seizure at 24 hours. None of the patients had early malignant edema | “BP excursions” (greater than 185 SBP or greater than 110 DB) associated with END | NR |
| Yamamoto, 2011a | N=394 patients with penetrating artery territory infarcts in the territories of the  lenticulostriate arteries and anterior pontine arteries; small infarcts no greater than 20 mmin diameter  Radiologically defined BAD of the lenticulostriate  arteries was defined as infarcts with size more than 10 mm in diameter on axial slice and visible for 3 ormore  axial slices, and that of the anterior pontine arteries was defined as unilateral infarcts extending to the basal  surface of the pons  2002-2008, Kyoto, includes patients also described in Yamamoto 2010 and 2011 | Worsening by ≥1-point in the NIHSS for motor function during the first 5 days | Overall 95/394 (24.1%)  57/263 (21.7%) for lenticulostriate  38/131 (29%) for anterior pontine | NR | Initial NIHSS, incidence of progressive motor deficits and poor functional outcome were significantly higher in patients with BAD | Yes. mRS≥3 higher in patients with END |
| Yamamoto, 2014 | N=110 patients with small vessel occlusion (TOAST) within 2 days from onset of acute ischemic stroke, MRI confirmed, no upper size limit. April 2008-July 2012 Tokushima, not Kyoto | 1 point or more worsening of the NIHSS score during  hospitalization | 32/110 (29%) | NR | White matter hyperintensities and lack of previous stroke independently associated with END | NR |
| Yu, 2015 | N=75 patients with acute “lacunae-sized infarctions” in the perforating arterial territory (pons or territory of the lenticulostriate arteries), which was confirmed by cranial magnetic resonance image (MRI). | Any increase in the NIHSS score in the first 7 days after stroke onset | 30/75 (40%) | NR | “satellite lesions” on DWI associated with END | NR |
| Zhang, 2018 | N=411 patients with first-episode lacunar infarcts and hypertension within 24 hours of symptom onset | Worsening by ≥1 point in the National Institute of Health Stroke Scale (NIHSS) for motor function | 80/411 (19.5%) | NR | Combination therapy with CCB, ACEI/ARB, and beta-blockers had the lowest risk of deterioration | NR |

**Additional references of studies included in the meta-analysis (main paper, Figure 2).**

1. Serena J, Leira R, Castillo J, et al. Neurological deterioration in acute lacunar infarctions: the role of excitatory and inhibitory neurotransmitters. Stroke. 2001;32(5):1154-61.
2. Kim YS, Lee KY, Koh SH, et al. The role of matrix metalloproteinase 9 in early neurological worsening of acute lacunar infarction. European neurology. 2006;55(1):11-5.
3. Hwang YH, Seo JG, Lee HW, et al. Early neurological deterioration following intravenous recombinant tissue plasminogen activator therapy in patients with acute lacunar stroke. Cerebrovascular Diseases. 2008;25(4):355-9.
4. Cho KH, Kang DW, Kwon SU, et al. Lesion volume increase is related to neurologic progression in patients with subcortical infarction. Journal of the neurological sciences. 2009;284(1-2):163-7.
5. Koh SH, Park CY, Kim MK, Lee KY, et al. Microbleeds and free active MMP‐9 are independent risk factors for neurological deterioration in acute lacunar stroke. European journal of neurology. 2011;18(1):158-64.
6. Kwon HM, Lim JS, Park HK, et al. Hypertriglyceridemia as a possible predictor of early neurological deterioration in acute lacunar stroke. Journal of the neurological sciences. 2011;309(1-2):128-30.
7. Ryu DW, Shon YM, Kim BS, et al. Conglomerated beads shape of lacunar infarcts on diffusion-weighted MRI: what does it suggest? Neurology. 2012;78(18):1416-9.
8. Sakamoto Y, Kimura K, Aoki J, et al. The augmentation index as a useful indicator for predicting early symptom progression in patients with acute lacunar and atherothrombotic strokes. Journal of the neurological sciences. 2012;321(1-2):54-7.
9. Griebe M, Fischer E, Kablau M, et al. Thrombolysis in patients with lacunar stroke is safe: an observational study. Journal of neurology. 2014;261(2):405-11.
10. Nakase T, Yoshioka S, Sasaki M, Suzuki A. Clinical evaluation of lacunar infarction and branch atheromatous disease. Journal of Stroke and Cerebrovascular Diseases. 2013;22(4):406-12.
11. Feng C, Xu Y, Hua T, et al. Irregularly shaped lacunar infarction: risk factors and clinical significance. Arquivos de neuro-psiquiatria. 2014;71:769-73.
12. Umemura T, Senda J, Fukami Y, et al. Impact of albuminuria on early neurological deterioration and lesion volume expansion in lenticulostriate small infarcts. Stroke. 2014;45(2):587-90.
13. Vahidy FS, Hicks WJ, Acosta I, et al. Neurofluctuation in patients with subcortical ischemic stroke. Neurology. 2014;83(5):398-405.
14. Jeong SK, Lee JH, Nam DH, Kim JT, et al. Basilar artery angulation in association with aging and pontine lacunar infarction: a multicenter observational study. Journal of atherosclerosis and thrombosis. 2015;22(5):509-17.
15. Nakase T, Yamamoto Y, Takagi M, et al. The impact of diagnosing branch atheromatous disease for predicting prognosis. Journal of Stroke and Cerebrovascular Diseases. 2015;24(10):2423-8.
16. Yu YP, and Tan L. The infarct shape predicts progressive motor deficits in patients with acute lacunae-sized infarctions in the perforating arterial territory. Internal Medicine. 2015;54(23):2999-3004.
17. Takeuchi M, Miyashita K, Nakagawara J, et al. Analysis of factors associated with progression and long-term outcomes of penetrating artery territory infarction: a retrospective study. Journal of Stroke and Cerebrovascular Diseases. 2016;25(8):1952-9.
18. Chen Y, Wang A, Tang J, et al. Association of white matter integrity and cognitive functions in patients with subcortical silent lacunar infarcts. Stroke. 2015;46(4):1123-6.
19. Gökçal E, Niftaliyev E, Baran G, et al. Progressive deficit in isolated pontine infarction: the association with etiological subtype, lesion topography and outcome. Acta Neurologica Belgica. 2017;117(3):649-54.
20. Lee KJ, Jung H, Oh YS, et al. The fate of acute lacunar lesions in terms of shape and size. Journal of Stroke and Cerebrovascular Diseases. 2017;26(6):1254-7.
21. Shin J, Chung JW, Park MS, et al. Outcomes after ischemic stroke caused by intracranial atherosclerosis vs dissection. Neurology. 2018;91(19):e1751-9.
22. Naess H, Thomassen L, Waje‐Andreassen U, et al. High risk of early neurological worsening of lacunar infarction. Acta Neurologica Scandinavica. 2018;139(2):143-9.
23. Saji N, Tone S, Murotani K, Yagita Y, et al. Cilostazol may decrease plasma inflammatory biomarkers in patients with recent small subcortical infarcts: a pilot study. Journal of Stroke and Cerebrovascular Diseases. 2018;27(6):1639-45.
24. Zhang J, Han F, Liang X, Li M, et al. Lacune and large perivascular space: two kinds of cavities are of different risk factors and stroke risk. Cerebrovascular Diseases. 2020;49(5):522-30.
25. Im SH, Jang DK, Kim H, Park SK, et al. Long-term mortality in patients with moyamoya angiopathy according to stroke presentation type in South Korea. Acta Neurochirurgica. 2021;163(12):3473-81.
26. Bashir S, Terceño M, Buxó M, Silva Y, et al. Progressive Lacunar Strokes: A Predictive Score. Journal of Stroke and Cerebrovascular Diseases. 2022;31(8):106510.
27. Farooqui A, Albayram MS, Reddy VB, et al. Neurological deterioration and computed tomography perfusion changes with increased time to peak in lacunar stroke. Brain Circulation. 2022;8(1):17-23.
28. Lin Q, Ye T, Ye P, Borghi C, et al. Hypertension in stroke survivors and associations with national premature stroke mortality: data for 2· 5 million participants from multinational screening campaigns. The Lancet Global Health. 2022;10(8):e1141-9.
29. Pan Y, Meng X, Jin A, Johnston SC, Li H, et al. Time course for benefit and risk with ticagrelor and aspirin in individuals with acute ischemic stroke or transient ischemic attack who carry CYP2C19 loss-of-function alleles: a secondary analysis of the CHANCE-2 randomized clinical trial. JAMA neurology. 2022;79(8):739-45.
30. Tan C, Zhao L, Dai C, et al. Risk factors related to early neurological deterioration in lacunar stroke and its influence on functional outcome. International Journal of Stroke. 2023;18(6):681-8.
31. Wei C, Shen T, Tang X, et al. Cerebral small vessel disease combined with cerebral collaterals to predict the prognosis of patients with acute large artery atherosclerotic stroke. Frontiers in Neurology. 2022;13:969637.
32. Forti P, Maioli F. The Prognostic Significance of Early Glycemic Profile in Acute Ischemic Stroke Depends on Stroke Subtype. Journal of Clinical Medicine. 2023;12(5):1794.
33. Nam KW, Kwon HM, and Lee YS. Infarct growth velocity predicts early neurological outcomes in single subcortical infarction. Scientific Reports. 2023;13(1):4511.

# PRISMA 2020 Main Checklist

| **Topic** | **No.** | **Item** | **Location where item is reported** |
| --- | --- | --- | --- |
| **TITLE** |  |  |  |
| **Title** | 1 | Identify the report as a systematic review. | Title |
| **ABSTRACT** |  |  |  |
| **Abstract** | 2 | See the PRISMA 2020 for Abstracts checklist |  |
| **INTRODUCTION** |  |  |  |
| **Rationale** | 3 | Describe the rationale for the review in the context of existing knowledge. | Page 4 |
| **Objectives** | 4 | Provide an explicit statement of the objective(s) or question(s) the review addresses. | Page 4 |
| **METHODS** |  |  |  |
| **Eligibility criteria** | 5 | Specify the inclusion and exclusion criteria for the review and how studies were grouped for the syntheses. | Page 5 |
| **Information sources** | 6 | Specify all databases, registers, websites, organisations, reference lists and other sources searched or consulted to identify studies. Specify the date when each source was last searched or consulted. | Page 5 |
| **Search strategy** | 7 | Present the full search strategies for all databases, registers and websites, including any filters and limits used. | Page 5 |
| **Selection process** | 8 | Specify the methods used to decide whether a study met the inclusion criteria of the review, including how many reviewers screened each record and each report retrieved, whether they worked independently, and if applicable, details of automation tools used in the process. | Page 5 |
| **Data collection process** | 9 | Specify the methods used to collect data from reports, including how many reviewers collected data from each report, whether they worked independently, any processes for obtaining or confirming data from study investigators, and if applicable, details of automation tools used in the process. | Page 5 |
| **Data items** | 10a | List and define all outcomes for which data were sought. Specify whether all results that were compatible with each outcome domain in each study were sought (e.g. for all measures, time points, analyses), and if not, the methods used to decide which results to collect. | Page 5 |
|  | 10b | List and define all other variables for which data were sought (e.g. participant and intervention characteristics, funding sources). Describe any assumptions made about any missing or unclear information. | Page 5 |
| **Study risk of bias assessment** | 11 | Specify the methods used to assess risk of bias in the included studies, including details of the tool(s) used, how many reviewers assessed each study and whether they worked independently, and if applicable, details of automation tools used in the process. | Not done |
| **Effect measures** | 12 | Specify for each outcome the effect measure(s) (e.g. risk ratio, mean difference) used in the synthesis or presentation of results. | Not applicable |
| **Synthesis methods** | 13a | Describe the processes used to decide which studies were eligible for each synthesis (e.g. tabulating the study intervention characteristics and comparing against the planned groups for each synthesis (item 5)). | Not done |
|  | 13b | Describe any methods required to prepare the data for presentation or synthesis, such as handling of missing summary statistics, or data conversions. | Not applicable |
|  | 13c | Describe any methods used to tabulate or visually display results of individual studies and syntheses. | Page 5 |
|  | 13d | Describe any methods used to synthesize results and provide a rationale for the choice(s). If meta-analysis was performed, describe the model(s), method(s) to identify the presence and extent of statistical heterogeneity, and software package(s) used. | Page 5 |
|  | 13e | Describe any methods used to explore possible causes of heterogeneity among study results (e.g. subgroup analysis, meta-regression). | Page 5 |
|  | 13f | Describe any sensitivity analyses conducted to assess robustness of the synthesized results. | Page 5 |
| **Reporting bias assessment** | 14 | Describe any methods used to assess risk of bias due to missing results in a synthesis (arising from reporting biases). | Not done |
| **Certainty assessment** | 15 | Describe any methods used to assess certainty (or confidence) in the body of evidence for an outcome. | Not done |
| **RESULTS** |  |  |  |
| **Study selection** | 16a | Describe the results of the search and selection process, from the number of records identified in the search to the number of studies included in the review, ideally using a flow diagram. | Figure 1 |
|  | 16b | Cite studies that might appear to meet the inclusion criteria, but which were excluded, and explain why they were excluded. | Figure 1 |
| **Study characteristics** | 17 | Cite each included study and present its characteristics. | Supplementary Table 1 |
| **Risk of bias in studies** | 18 | Present assessments of risk of bias for each included study. | Not done |
| **Results of individual studies** | 19 | For all outcomes, present, for each study: (a) summary statistics for each group (where appropriate) and (b) an effect estimate and its precision (e.g. confidence/credible interval), ideally using structured tables or plots. | Figure 1 |
| **Results of syntheses** | 20a | For each synthesis, briefly summarise the characteristics and risk of bias among contributing studies. | Not done |
|  | 20b | Present results of all statistical syntheses conducted. If meta-analysis was done, present for each the summary estimate and its precision (e.g. confidence/credible interval) and measures of statistical heterogeneity. If comparing groups, describe the direction of the effect. | Page 5, Figure 1 |
|  | 20c | Present results of all investigations of possible causes of heterogeneity among study results. | Page 6 |
|  | 20d | Present results of all sensitivity analyses conducted to assess the robustness of the synthesized results. | Page 6 |
| **Reporting biases** | 21 | Present assessments of risk of bias due to missing results (arising from reporting biases) for each synthesis assessed. | Not done |
| **Certainty of evidence** | 22 | Present assessments of certainty (or confidence) in the body of evidence for each outcome assessed. | Not applicable |
| **DISCUSSION** |  |  |  |
| **Discussion** | 23a | Provide a general interpretation of the results in the context of other evidence. | Page 9 |
|  | 23b | Discuss any limitations of the evidence included in the review. | Discussion |
|  | 23c | Discuss any limitations of the review processes used. | Not done |
|  | 23d | Discuss implications of the results for practice, policy, and future research. | Discussion |
| **OTHER INFORMATION** |  |  |  |
| **Registration and protocol** | 24a | Provide registration information for the review, including register name and registration number, or state that the review was not registered. | Not applicable |
|  | 24b | Indicate where the review protocol can be accessed, or state that a protocol was not prepared. | Not applicable |
|  | 24c | Describe and explain any amendments to information provided at registration or in the protocol. | Not applicable |
| **Support** | 25 | Describe sources of financial or non-financial support for the review, and the role of the funders or sponsors in the review. | Page 14 |
| **Competing interests** | 26 | Declare any competing interests of review authors. | Page 14 |
| **Availability of data, code and other materials** | 27 | Report which of the following are publicly available and where they can be found: template data collection forms; data extracted from included studies; data used for all analyses; analytic code; any other materials used in the review. | Not applicable |

#####

# PRIMSA Abstract Checklist

| **Topic** | **No.** | **Item** | **Reported?** |
| --- | --- | --- | --- |
| **TITLE** |  |  |  |
| **Title** | 1 | Identify the report as a systematic review. | Yes |
| **BACKGROUND** |  |  |  |
| **Objectives** | 2 | Provide an explicit statement of the main objective(s) or question(s) the review addresses. | Yes |
| **METHODS** |  |  |  |
| **Eligibility criteria** | 3 | Specify the inclusion and exclusion criteria for the review. | No |
| **Information sources** | 4 | Specify the information sources (e.g. databases, registers) used to identify studies and the date when each was last searched. | Yes |
| **Risk of bias** | 5 | Specify the methods used to assess risk of bias in the included studies. | No |
| **Synthesis of results** | 6 | Specify the methods used to present and synthesize results. | Yes |
| **RESULTS** |  |  |  |
| **Included studies** | 7 | Give the total number of included studies and participants and summarise relevant characteristics of studies. | Yes |
| **Synthesis of results** | 8 | Present results for main outcomes, preferably indicating the number of included studies and participants for each. If meta-analysis was done, report the summary estimate and confidence/credible interval. If comparing groups, indicate the direction of the effect (i.e. which group is favoured). | Yes |
| **DISCUSSION** |  |  |  |
| **Limitations of evidence** | 9 | Provide a brief summary of the limitations of the evidence included in the review (e.g. study risk of bias, inconsistency and imprecision). | Yes |
| **Interpretation** | 10 | Provide a general interpretation of the results and important implications. | Yes |
| **OTHER** |  |  |  |
| **Funding** | 11 | Specify the primary source of funding for the review. | Yes |
| **Registration** | 12 | Provide the register name and registration number. | No |

*From:* Page MJ, McKenzie JE, Bossuyt PM, Boutron I, Hoffmann TC, Mulrow CD, et al. The PRISMA 2020 statement: an updated guideline for reporting systematic reviews. MetaArXiv. 2020, September 14. DOI: 10.31222/osf.io/v7gm2. For more information, visit: <www.prisma-statement.org>
